# Comparison of Chemical Constituents in Pseudostellariae Radix with Different Dosage Forms Based on HPLC-Q-Exactive Orbitrap/MS Combined with Multivariate Statistical Analysis

**DOI:** 10.1155/2021/6644127

**Published:** 2021-05-08

**Authors:** Yujiao Hua, Xiaoyuan Liu, Fen Xie

**Affiliations:** Department of Clinical Pharmacy, Affiliated Hospital of Jiangnan University, Wuxi 214000, China

## Abstract

**Background:**

Pseudostellariae Radix (PR) is an important traditional Chinese herbal medicine with vast clinical consumptions, which has two different dosage forms, PR decoction pieces and PR formula granules. However, these two forms are bound to have an impact on the accumulation of the effective components in PR, so the effectiveness of clinical use cannot be guaranteed.

**Objective:**

To determine the effective composition of PR.

**Methods:**

In this research, PR decoction pieces and formula granules were collected, and their composition was detected by HPLC-Q-Exactive Orbitrap/MS; multivariate statistical analysis was used to distinguish differential metabolites between PR decoction pieces and formula granules.

**Results:**

A clear cut difference in the composition of the two groups was observed. 98 differential chemical constituents could be identified in the positive mode, while 52 differential chemical compositions could be identified in the negative mode. The differential chemical compositions were mainly concentrated in flavonoids, organic acids, fatty acids, and amino acids and present different change rules, mainly involved in the isoquinoline alkaloid biosynthesis metabolic pathways.

**Conclusions:**

This study provides basic information to reveal the influence law of different dosage forms on the metabolite synthesis and quality formation mechanism of PR.

## 1. Introduction

Pseudostellariae Radix (PR) is an arid tuberous root of *P. heterophylla* (Miq.) Pax ex Pax et Hoffm [1]. It is a kind of important traditional Chinese herbal medicine which has the functions of promoting immunity, relieving cough, antitumor, antioxidation, and protecting myocardial function [2]. This herbal medicine is used wildly for its positive effects. It is reported that PR can be used for inappetence [3], thirst [4], debility [5], diabetes [6], and weakness after illness [7]. Currently, PR decoction pieces and formula granules are utilized in a Chinese hospital; among them, PR formula granules is a novel form of decoction pieces, which is prepared by water extraction, concentration, drying, and granulation of the PR decoction pieces [8, 9]. These two types of PR used have their own advantages and disadvantages. One of the advantages of PR decoction pieces being that it has been prescribed since thousands of years as a potent drug with high traditional following. On the other hand, the advantages of PR formula granules lie in small package, multiple varieties, and multiple specifications, and the relevant parameters of drugs are clearly marked, which is conducive to the identification and selection of medicinal materials by patients; also, the formula granules are easy for patients to take home [10, 11].

The different dosage forms are bound to have an impact on the accumulation of its effective constituents, so the effectiveness of clinical use cannot be guaranteed, and the active components of these two dosage forms have not been studied in depth. In order to have a more comprehensive understanding on the synthesis and accumulation of metabolites in PR with different dosage forms, as well as the difference in quality between PR decoction pieces and formula granules, it is necessary to build a quality analysis method for the overall chemical constituents of PR with different dosage forms.

Metabolomics technology is a new omics technology developed in the mid-1990s, among which plant metabolomics is a high-throughput, unbiased, and comprehensive analysis technology for metabolomics in plant extracts, and it is especially fit for the analysis of multicomponent complex systems of TCM [12–14]. In recent years, liquid-mass coupling technology is widely used in the qualitative and quantitative research of complex TCM systems [15, 16], and the commonly used mass spectrometry techniques include quadrupole mass spectrometry, ion trap mass spectrometry, time-of-flight mass spectrometry, and compound tandem mass spectrometry [17–20]. The structure of the compounds can be rapidly identified based on the fragmentation information of the multistage mass spectrometry compounds and related database. Liquid phase with high efficiency and rapid separation performance combined with sensitive and accurate mass spectrometry is one of the most promising metabolomics technologies and has been widely used in the analysis of polar compounds, thermal unstable compounds, and macromolecular compounds.

In this study, an accurate and reliable method based on HPLC-Q-Exactive Orbitrap/MS coupled with multivariate statistical analysis and signal pathway analysis has been developed to analyze the differential chemical compositions and major metabolic pathways in PR decoction pieces and formula granules, exploring the dynamic change law of different dosage forms of PR. It is expected to provide basic data for revealing the influence of different dosage forms on the synthesis and accumulation of metabolites of PR and for discussing the formation mechanism of the quality in PR decoction pieces and formula granules.

## 2. Materials and Methods

### 2.1. Chemicals and Reagents

Formic acid and acetonitrile of HPLC grade were purchased from Sigma-Aldrich (St. Louis, MO, USA) and Merck (Darmstadt, Germany), respectively. Ultrapure water was prepared using a Milli-Q purifying system (Millipore, Bedford, MA, USA) under a resistivity of 18.2 MΩ/cm, and other reagent solutions such as methanol and 2-chloro-L-phenylalanine were analytical grade (Merck, Darmstadt, Germany).

### 2.2. Plant Materials

Five PR decoction pieces were purchased from Suzhou Tianling Chinese Herbal Medicine Co. Ltd., Jiangsu Province, China. Five PR formula granules were purchased from Jiangyin Tianjiang Pharmaceutical Co. Ltd., Jiangsu Province, China. The botanical origin of the materials was identified by Professor Xunhong Liu (Department for Authentication of Chinese Medicines, Nanjing University of Chinese Medicine, China), and the voucher specimens were deposited at TCM pharmacy of Affiliated Hospital of Jiangnan University.

### 2.3. Sample Preparations

PR decoction pieces and PR formula granules were naturally put at room temperature prior to HPLC-Q-Exactive Orbitrap/MS analysis. The dried PR decoction pieces and formula granules were pulverized into homogeneous powders (80 mesh). Powder samples (0.1 g) were accurately weighed out and transferred to a 25 mL conical flask equipped with a stopper. Then, samples were applied to extraction procedure, extracted with 800 *μ*L of methanol and 10 *μ*L of internal standard (2.8 mg/mL, DL-o-chlorophenylalanine). All samples were grinded to fine powder using a grinding mill at 65 Hz for 90 s, and then, they were ultrasonicated for 30 min, by 40 KHz and let stand for 1 hour at −20°C. The samples were centrifuged at 12000 rpm and 4°C for 15 min, and the supernatants were filtered through a 0.22 *μ*m membrane before injection into the HPLC system.

### 2.4. HPLC-Q-Exactive Orbitrap/MS Conditions

The HPLC analysis was performed on the Ultimate 3000 HPLC system (Thermo, Waltham, MA, USA). A hyper gold C18 column (100 mm × 2.1 mm × 1.9 *μ*m) (Thermo, Waltham, MA, USA) was used to carry out the chromatographic separation at 40°C. The mobile phase consisted of eluent A (water + 5% acetonitrile + 0.1% formic acid, *v/v*) and eluent B (acetonitrile + 0.1% formic acid, *v/v*) with a flow rate of 0.35 mL/min with a liner gradient program: 100%–80% A from 0 to 1.5 min, 80%–0% A from 1.5 to 9.5 min, 0% A from 9.5 to 14.5 min, 0%–100% A from 14.5 to 14.6 min, and 100% A from 14.6 to 18 min.

Mass spectrometry detection was performed on Q-Exactive Orbitrap/MS (Thermo, Waltham, MA, USA) equipped with an electrospray ionization (ESI) source operating in the positive and negative ion modes. The scan range was m/z 50–1000 and m/z 50–1100 in positive and negative ion modes, respectively. The optimized instrumental parameters were as follows: heater temperature was 300°C, sheath gas flow rate was 45 arb, Aux gas flow rate was 15 arb, sweep gas flow rate was 1 arb, and capillary temperature was 350°C. The spray voltage was floating at 3.0 KV (ESI^+^) or 3.2 KV (ESI^−^). S-lens RF level was 30% (ESI^+^) or 60% (ESI^−^).

### 2.5. Data Analysis

The data were performed feature extraction and preprocessed with Compound Discoverer software (Thermo, Waltham, MA, USA) and then normalized and edited into a two-dimensional data matrix by Excel 2010 software (Redmond, WA, USA), including retention time (RT), compound molecular weight (compMW), observations (samples), and peak intensity. The edited data matrix was imported into SIMCA-P 13.0 software (Umetrics, Umea, Sweden) for multivariate statistical analysis. Principal component analysis (PCA) was performed to intuitively express the difference in chemical compositions between PR decoction pieces and PR formula granules by observing the aggregation of each sample. Partial least squares discriminant analysis (PLS-DA) and orthogonal least squares discriminant analysis (OPLS-DA) were performed to further classify the samples. Two parameters, *R*^2^*Y* and *Q*^2^, were used to evaluate the model, the closer R^2^Y is to 1, the more stable the model is, and *Q*^2^ > 0.5 indicates a high prediction rate. Variable importance in the projection (VIP) > 1 via OPLS-DA analysis and the *p* value of the *t*-test (*p* < 0.05) were used to find potential metabolites that significantly contributed to the difference among the groups [21]. Biochemical databases, METLIN (http://metlin.scripps.edu/), HMDB (http://www.hmdb.ca/), KEGG (http://www.kegg.com/), and MetaboAnalyst (http://www.metaboanalyst.ca/), were used to identify potential metabolites. According to the data of MetaboAnalyst, the impact value threshold was set at 0.6, and therefore, the most important potential metabolic pathways were filtered out [22].

## 3. Results and Discussion

### 3.1. Based Peak Chromatogram

The extraction conditions, including extraction (ultrasonic and refluxing extraction), extraction solvent (100% methanol, 70% methanol, 50% methanol, and 30% methanol), and extraction time (15, 30, 45, 60, and 75 min) were optimized to acquire the most outstanding extraction efficiency. The results showed that the chromatographic peak shape and relative peak area were superior to the others under ultrasonic extraction with 100% methanol for 30 min.

In order to gain fast effective analysis, a hyper gold C18 column (100 mm × 2.1 mm × 1.9 *μ*m) column was employed for this experiment. Different mobile phases (including methanol-water, acetonitrile-water, methanol-0.1% formic acid water, acetonitrile-0.1% formic acid water, acetonitrile with 0.1% formic acid water-acetonitrile with 0.1% formic acid, and 5% acetonitrile with 0.1% formic acid water-acetonitrile with 0.1% formic acid), flow rate (0.25, 0.35, and 0.45 mL/min), and column temperature (35, 40, and 45°C) were examined and compared. As a result, a 5% acetonitrile with 0.1% formic acid water-acetonitrile with 0.1% formic acid at a flow rate of 0.35 mL/min and a column temperature of 40°C was found satisfactory for separation of molecules in a short time.

**Figure 1:**
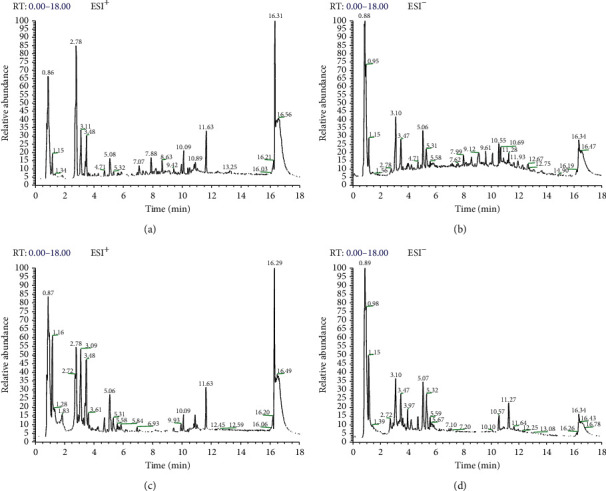
BPC of PR decoction pieces (a-b) and PR formula granules (c-d) in both positive and negative modes.

According to the set conditions of sample treatment, liquid chromatography and mass spectrometry were determined. The based peak chromatogram (BPC) of PR decoction pieces and formula granules obtained from the analysis in both positive and negative modes is shown in Figure 1.

### 3.2. Multivariate Statistical Analysis

#### 3.2.1. Principal Component Analysis

**Figure 2:**
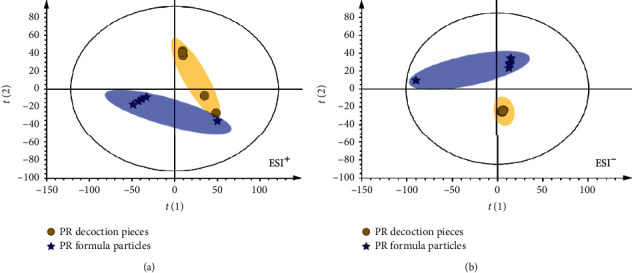
The scores plot obtained from PCA analysis of PR decoction pieces and formula granules. The scores plot of PCA revealed that the 10 samples were classified into two major groups in the positive mode (a) and negative mode (b).

Based on LC-MS spectra, chemical classification of all samples was carried out by multivariate data analysis, which aims to evaluate the differences in chemical constituents of PR decoction pieces (TY) and formula granules (TK). Principal component analysis (PCA) was utilized to reduce the dimensions of multivariate problems. After Vilfredo Pareto with mean centering, the data were showed as scores in a coordinate system of latent variables, which resulted from the above samples. The PCA score plot (R^2^X = 0.805, *Q*^2^ = 0.686 in ESI^+^, Figure 2(a); *R*^2^*X* = 0.886, and *Q*^2^ = 0.654 in ESI^−^, Figure 2(b)) showed clear separation in different dosage forms of PR in both ESI^+^ and ESI^−^. This indicated that there was a significant difference in chemical constituents between TY and TK.

#### 3.2.2. Partial Least Square Discriminant Analysis

**Figure 3:**
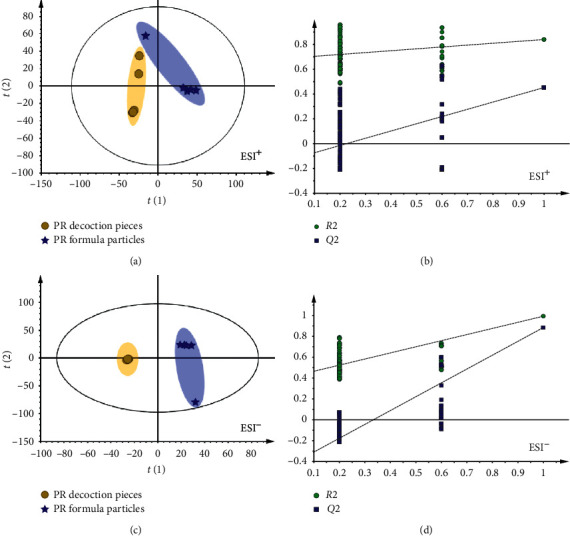
PLS-DA scores plot and the permutation test with 200 permutations of PR decoction pieces and formula granules. The scores plot of PLS-DA revealed that PR decoction pieces and PR formula granules could be clearly distinguished into two groups in the positive mode (a) and negative mode (c). The permutation test with 200 permutations indicated that PLS-DA is effective and can be used for subsequent differential component analysis in the positive mode (b) and negative mode (d).

Partial least square discriminant analysis (PLS-DA) is a supervised analysis, which extends a regression of PCA and uses class information to maximize the separation between groups of observations [23]. This frequently used classification method is categorical (categories described with dummy variables) and expressed the class membership of the statistical units. In this research, the model parameters were *R*^2^*X* = 0.799, *R*^2^*Y* = 0.840, *Q*^2^ = 0.452 in ESI^+^ (Figure 3(a)); *R*^2^*X* = 0.884, *R*^2^*Y* = 0.993, and *Q*^2^ = 0.885 in ESI^−^ (Figure 3(c)). The PLS-DA scores plot showed that TY and TK were clearly isolated into two groups, and the interclass differences were less than that in the PCA model, so the PCA model was more effective to ensure the differences in two different dosages of PR. The PLS-DA model was further validated by a permutation test with 200 permutations (Figures 3(b) and 3(d)). The *R*^2^ and *Q*^2^ values generated by the random permutation at the left end are both smaller than the original values at the right end, indicating that the predictive power of the original model is greater than that of the random permutation *y* variables, so the model is effective and can be used for subsequent differential component analysis [24].

#### 3.2.3. Orthogonal Least Squares Discriminant Analysis

**Figure 4:**
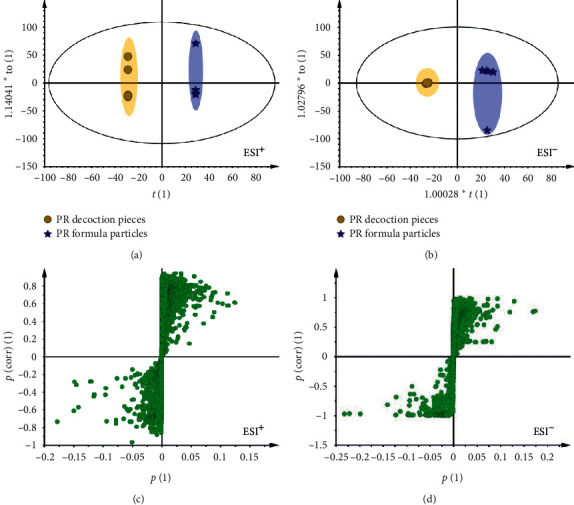
OPLS-DA scores plot and S-plot of PR decoction pieces and formula granules. The scores plot of OPLS-DA revealed that PR decoction pieces and PR formula granules were significantly different in the two classes in the positive mode (a) and negative mode (b). S-plots indicated that the farther the distance from the central region represented the metabolites contributing more to the separation between groups in the positive mode (c) and negative mode (d).

In order to further identify the significant metabolites contributing to distinction in the two forms of PR, orthogonal least squares discriminant analysis (OPLS-DA), a supervised pattern recognition approach was performed [25]. In OPLS-DA scores plot, each spot represents a sample. As shown in Figure 4, the TY group can be clearly separated from the TK group in both positive and negative modes, and the model parameters were *R*^2^*X* = 0.933, *R*^2^*Y* = 1, *Q*^2^ = 0.673 in ESI^+^ (Figure 4(a)); *R*^2^*X* = 0.884, *R*^2^*Y* = 0.993, and *Q*^2^ = 0.984 in ESI^−^ (Figure 4(b)), which indicated good ability of prediction and reliability of the model. To identify the metabolites contributing to the discrimination, S-plots were generated (Figure 4(c) and 4(d)). Each spot in OPLS-DA scores plot and S-plots represents a variance. Farther the distance from the central region, higher is the contribution of the metabolites. The importance of each variance to classification was determined by the value of variable in the projection (VIP). Metabolites with the VIP value above 1.0 and *P* value below 0.05 were considered as potential metabolic markers.

### 3.3. Identification of Differential Compositions with Relative Contents Analysis

**Figure 5:**
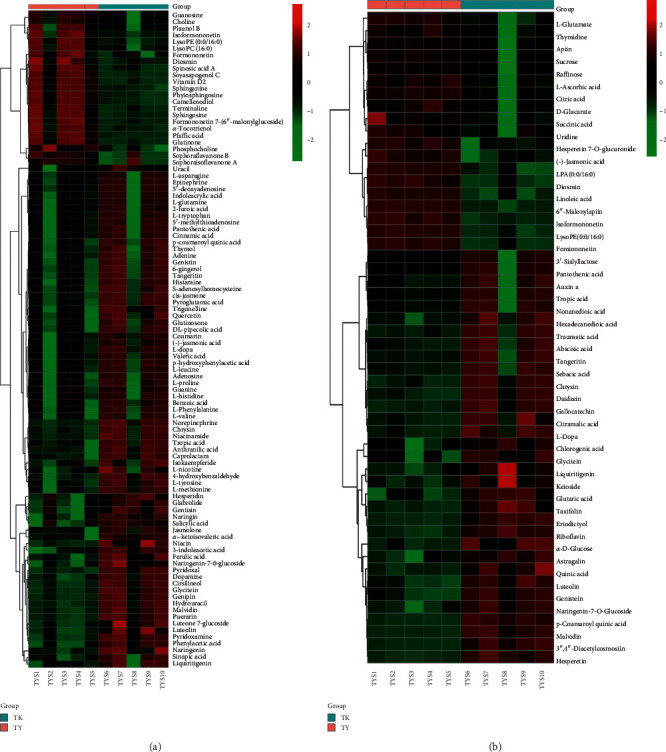
Hierarchical clustering analysis of PR decoction pieces and formula granules. Clear differentiation was observed in the heat map, colors varying from green to red graphically indicated that the relative contents of metabolites were from low to high. 98 and 52 differential chemical compositions were identified in the positive mode (a) and negative mode (b).

VIP >1 of OPLS-DA combined with the *t*-test (*p* < 0.05) was used to discover the significantly differential metabolites in TY and TK. METLIN and HMDB databases are used to search the accurate mass-to-charge ratio to identify the differential chemical compositions. As seen from hierarchical clustering analysis (Figures 5(a) and 5(b)), a total of 98 and 52 differential chemical compositions were identified in the positive mode and negative mode, respectively, and the detailed information is given in Table S1 and Table S2.

Clear differentiation was observed in the heat map based on differential chemical compositions, and colors varying from green to red graphically indicate that the relative contents of metabolites are from low to high. In the positive mode, there were 23 differential chemical compositions that had the relative contents of TY greater than that in TK, while there were 75 differential chemical compositions that the relative contents of TY were less than that in TK. In the negative mode, there were 19 differential chemical compositions that the relative contents of TY were greater than that in TK, while there were 33 differential chemical compositions that the relative contents of TY were less than that in TK.

**Figure 6:**
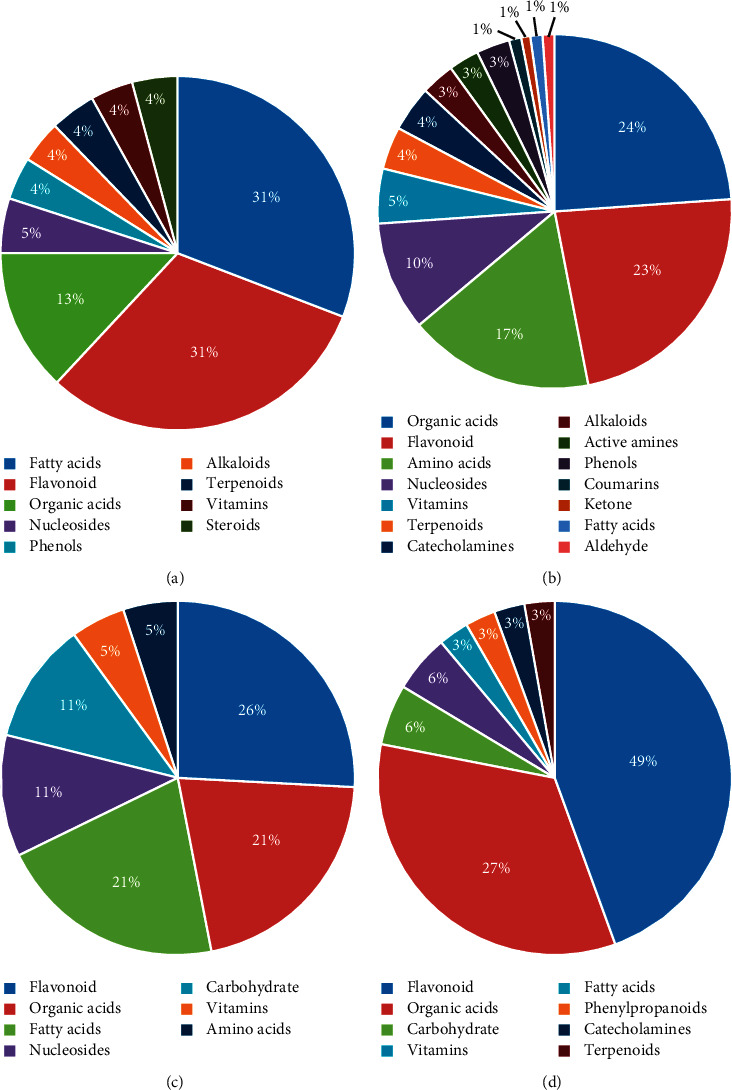
Comparative analysis of contents of differential chemical compositions in PR decoction pieces and formula granules. (a) The relative contents of differential chemical compositions in PR decoction pieces > PR formula granules in the positive mode. (b) The relative contents of differential chemical compositions in PR decoction pieces < PR formula granules in the positive mode. (c) The relative contents of differential chemical compositions in PR decoction pieces > PR formula granules in the negative mode. (d) The relative contents of differential chemical compositions in PR decoction pieces < PR formula granules in the negative mode.

As shown in Figures 6(a) and 6(b), in the positive mode, the relative contents of differential chemical compositions in TY above that in TK were distributed in nine kinds of compositions. Flavonoids and fatty acids had the highest proportion, accounting for 31%. The relative contents of differential chemical compositions in TY below that in TK were distributed in fourteen varieties of compositions. Among them, 18 organic acids accounted for the largest proportion (24%), followed by flavonoids (17, 23%) and amino acids (13, 17%). In the negative mode, the relative contents of differential chemical compositions in TY above that in TK were mainly involved in seven varieties of compositions. Flavonoids (5, 26%) were the most abundant, followed by organic acids (4, 21%) and fatty acids (4, 21%) (Figure 6(c)). The relative contents of differential chemical compositions in TY below that in TK belonged to eight varieties of compositions. Among them, flavonoids accounted for the highest proportion (16, 49%), followed by organic acids 9, 27% (Figure 6(d)). The results showed that differential chemical compositions of PR with different dosage forms were mainly concentrated in flavonoids, organic acids, amino acids, and fatty acids. In additional, in both positive and negative modes, the amounts and types of differential chemical compositions with higher relative contents in TK were both more than those with higher relative contents in TY, for example, flavonoids with antitumor effects, including chrysin, naringin, luteolin, and genistin; antimicrobial resistance to oxidation of organic compounds, including cinnamic acid, ferulic acid, and salicylic acid; and amino acid compounds with immune-promoting effects, including L-tryptophan, L-phenylalanine, and L-tyrosine.

### 3.4. Metabolic Pathway Analysis

**Figure 7:**
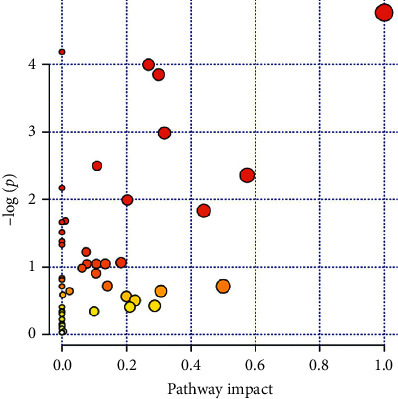
Analysis of pathway impact. Isoquinoline alkaloid biosynthesis which was the potential key metabolic pathway.

The metabolic pathway was analyzed by MetaboAnalyst website, and the Kyoto Encyclopedia of Genes and Genomes (KEGG) of the above identified compounds was introduced to MetaboAnalyst for pathway analysis. The radius of dots represents the impact value of the metabolic pathway. The larger the impact value, the larger the radius. The color of dots represents the *p* value of the metabolic pathway, and the lower the *p* value, the redder the color. The threshold value for the effect of the metabolic pathway was set to 0.6 with topological Fenix, greater than which will be selected as potential key metabolic pathways. Finally, one metabolic pathway, isoquinoline alkaloid biosynthesis (A), was coenriched (Figure 7).

## 4. Conclusions

In this research, the difference of chemical compositions in PR with different dosage forms was analyzed by HPLC-Q-Exactive Orbitrap/MS combined with multivariate statistical analysis. Results showed that the chemical compositions exited great differences in TY and TK. A total of 98 differential chemical compositions were found in ESI^+^, while 52 were found in ESI^−^. These constituents were mainly concentrated in flavonoids, organic acids, fatty acids, and amino acids, mainly involved in the isoquinoline alkaloid biosynthesis metabolic pathways. In addition, both in positive and negative modes, the amounts and types of differential chemical compositions with higher relative contents in TK were both more than those with higher relative contents in TY, including flavonoids with the antitumor effect, organic acids containing the antibacterial antioxidant effect, and amino acids which can boost immunity.

This study will provide the basic information for exploring the influence law of different dosage forms on the synthesis and accumulation of metabolites in PR and its quality-forming mechanism. It will also provide a reliable and accurate approach for the analysis of complex samples and identification of differential chemical compositions.

## Data Availability

The data used to support the findings of this study are available from the corresponding author upon request.

## Supplementary Materials

Supplementary Materials Table S1. Components identified with significant differences from Pseudostellariae Radix with different dosage forms in the positive ion mode (*p* < 0.05). Table S2. Components identified with significant differences from Pseudostellariae Radix with different dosage forms in the negative ion mode (*p* < 0.05).
