# Supplementary material for: Comparison of Chemical Constituents in Pseudostellariae Radix with Different Dosage Forms Based on HPLC-Q-Exactive Orbitrap/MS Combined with Multivariate Statistical Analysis
Source: Evid Based Complement Alternat Med. 2021 May 8;2021:6644127. doi: 10.1155/2021/6644127 (PMC8128553; doi:10.1155/2021/6644127)
Supplement: Supplementary Materials — Table S1. Components identified with significant differences from Pseudostellariae Radix with different dosage forms in the positive ion mode (p < 0.05). Table S2. Components identified with significant differences from Pseudostellariae Radix with different dosage forms in the negative ion mode (p < 0.05). [file 6644127.f1.zip › 6644127.f1/Table S1.docx]

**Table S1 Components identified with significant differences from Pseudostellariae Radix with different dosage form in positive ion mode（p＜0.05）**

| NO. | *t*_R_/min | Compound | formula | *m/z* | Fold change |
| --- | --- | --- | --- | --- | --- |
| 1 | 0.88 | Guanosine | C_10_H_13_N_5_O_5_ | 283.0917 | 2.32 |
| 2 | 10.15 | α-Tocotrienol | C_29_H_44_O_2_ | 424.3352 | 6.13 |
| 3 | 7.416 | Phytosphingosine | C_18_H_39_NO_3_ | 317.2941 | 5.60 |
| 4 | 5.418 | Isoformononetin | C_16_H_12_O_4_ | 268.0744 | 4.25 |
| 5 | 8.324 | LysoPE(0:0/16:0) | C_21_H_44_NO_7_P | 453.2873 | 4.68 |
| 6 | 10.543 | Camellenodiol | C_29_H_46_O_3_ | 442.3461 | 9.72 |
| 7 | 4.155 | Formononetin | C_16_H_12_O_4_ | 268.0746 | 1.39 |
| 8 | 9.515 | Sphingosine | C_18_H_37_NO_2_ | 299.2834 | 4.49 |
| 9 | 7.92 | Sphinganine | C_18_H_39_NO_2_ | 301.2992 | 6.27 |
| 10 | 4.033 | Soyasapogenol C | C_30_H_48_O_2_ | 440.3671 | 9.24 |
| 11 | 10.572 | Pfaffic acid | C_29_H_44_O_3_ | 440.3305 | 7.76 |
| 12 | 16.271 | Phosphocholine | C_5_H_14_NO_4_P | 183.0667 | 1.96 |
| 13 | 7.07 | Sophoraflavanone B | C_20_H_20_O_5_ | 340.1324 | 0.78 |
| 14 | 4.919 | Glutinone | C_30_H_48_O | 424.3721 | 7.73 |
| 15 | 10.769 | Vitamin D2 | C_28_H_44_O | 396.3405 | 7.77 |
| 16 | 8.562 | LysoPC(16:0) | C_24_H_50_NO_7_P | 495.3345 | 4.30 |
| 17 | 3.662 | Diosmin | C_28_H_32_O_15_ | 608.1767 | 2.44 |
| 18 | 4.436 | Formononetin 7-(6''-malonylglucoside) | C_25_H_24_O_12_ | 516.1290 | 5.64 |
| 19 | 8.043 | Terminaline | C_23_H_41_NO_2_ | 363.3152 | 4.99 |
| 20 | 0.852 | Choline | C_5_H_14_NO | 103.0992 | 1.51 |
| 21 | 7.095 | Sophoraisoflavanone A | C_21_H_24_O_6_ | 370.1432 | 0.60 |
| 22 | 3.405 | Plaunol B | C_20_H_20_O_6_ | 356.1231 | 3.55 |
| 23 | 8.221 | spinosic acid A | C_30_H_48_O_4_ | 472.3573 | 2.92 |
| 24 | 5.157 | S-Adenosylhomocysteine | C_14_H_20_N_6_O_5_S | 384.1223 | -0.88 |
| 25 | 2.608 | L-Tryptophan | C_11_H_12_N_2_O_2_ | 204.0904 | -2.54 |
| 26 | 5.300 | cis-Jasmone | C_11_H_16_O | 164.1206 | -3.91 |
| 27 | 5.323 | Thymol | C_10_H_14_O | 150.1049 | -2.10 |
| 28 | 2.716 | Indoleacrylic acid | C_11_H_9_NO_2_ | 187.0640 | -2.33 |
| 29 | 3.730 | Hesperidin | C_28_H_34_O_15_ | 610.1921 | -0.43 |
| 30 | 1.672 | L-Phenylalanine | C_9_H_11_NO_2_ | 165.0796 | -2.57 |
| 31 | 1.155 | Adenosine | C_10_H_13_N_5_O_4_ | 267.0976 | -2.33 |
| 32 | 3.560 | Sinapic acid | C_11_H_12_O_5_ | 224.0693 | -1.34 |
| 33 | 0.996 | L-Tyrosine | C_9_H_11_NO_3_ | 181.0746 | -2.08 |
| 34 | 3.575 | Naringenin | C_15_H_12_O_5_ | 272.0694 | -1.70 |
| 35 | 2.673 | 5'-Methylthioadenosine | C_11_H_16_N_5_O_7_PS | 297.0907 | -3.52 |
| 36 | 2.037 | Pantothenic Acid | C_9_H_17_NO_5_ | 219.1115 | -3.33 |
| 37 | 3.527 | p-Hydroxyphenylacetic acid | C_8_H_8_O_3_ | 152.0479 | -2.57 |
| 38 | 1.154 | Adenine | C_5_H_5_N_5_ | 135.0550 | -2.07 |
| 39 | 2.979 | Coumarin | C_9_H_6_O_2_ | 146.0373 | -2.23 |
| 40 | 1.157 | Guanine | C_5_H_5_N_5_O | 151.0500 | -1.59 |
| 41 | 1.163 | Norepinephrine | C_8_H_11_NO_3_ | 169.0745 | -3.19 |
| 42 | 4.314 | Luteolin | C_15_H_10_O_6_ | 286.0487 | -1.39 |
| 43 | 3.731 | Cirsilineol | C_18_H_16_O_7_ | 344.0908 | -2.78 |
| 44 | 1.010 | L-Dopa | C_9_H_11_NO_4_ | 197.0695 | -1.93 |
| 45 | 5.708 | (-)-Jasmonic acid | C_12_H_18_O_3_ | 210.1263 | -1.72 |
| 46 | 5.086 | Isokaempferide | C_16_H_12_O_6_ | 300.0645 | -2.41 |
| 47 | 3.719 | Dopamine | C_8_H_11_NO_2_ | 153.0795 | -3.69 |
| 48 | 6.131 | 6-Gingerol | C_17_H_26_O_4_ | 294.1841 | -2.61 |
| 49 | 2.726 | L-Glutamine | C_5_H_10_N_2_O_3_ | 146.0696 | -1.82 |
| 50 | 5.853 | Jasmolone | C_11_H_16_O_2_ | 180.1157 | -1.27 |
| 51 | 1.694 | Pyridoxamine | C_8_H_12_N_2_O_2_ | 168.0905 | -2.08 |
| 52 | 6.000 | Chrysin | C_15_H_10_O_4_ | 254.0588 | -4.81 |
| 53 | 4.863 | Malvidin | C_17_H_15_O_7_ | 330.0751 | -4.05 |
| 54 | 4.989 | Glycitein | C_16_H_12_O_5_ | 284.0695 | -4.41 |
| 55 | 3.456 | Genipin | C_11_H_14_O_5_ | 226.0850 | -4.04 |
| 56 | 3.703 | Tropic acid | C_9_H_10_O_3_ | 166.0636 | -3.05 |
| 57 | 1.005 | L-Methionine | C_5_H_11_NO_2_S | 149.0516 | -2.95 |
| 58 | 4.110 | Tangeritin | C_20_H_20_O_7_ | 372.1221 | -4.06 |
| 59 | 6.161 | Glutinosone | C_14_H_20_O_2_ | 220.1471 | -1.91 |
| 60 | 1.305 | L-Leucine | C_6_H_13_NO_2_ | 131.0952 | -2.17 |
| 61 | 1.816 | Cinnamic acid | C_9_H_8_O_2_ | 148.0531 | -2.27 |
| 62 | 0.973 | L-Proline | C_5_H_9_NO_2_ | 115.0639 | -1.79 |
| 63 | 1.157 | Pyroglutamic acid | C_5_H_7_NO_3_ | 129.0432 | -3.15 |
| 64 | 1.327 | Epinephrine | C_9_H_13_NO_3_ | 183.0903 | -3.35 |
| 65 | 2.893 | ferulic acid | C_10_H_10_O_4_ | 194.0588 | -1.28 |
| 66 | 0.892 | Trigonelline | C_7_H_7_NO_2_ | 137.0483 | -1.60 |
| 67 | 0.949 | L-Asparagine | C_4_H_8_N_2_O_3_ | 132.0541 | -1.73 |
| 68 | 1.259 | Niacin | C_6_H_5_NO_2_ | 123.0326 | -2.71 |
| 69 | 3.576 | Naringin | C_27_H_32_O_14_ | 580.1817 | -0.77 |
| 70 | 3.532 | Anthranilic acid | C_7_H_7_NO_2_ | 137.0483 | -5.05 |
| 71 | 2.892 | Salicylic acid | C_7_H_6_O_3_ | 138.0323 | -1.71 |
| 72 | 1.154 | DL-pipecolic acid | C_6_H_11_NO_2_ | 129.0796 | -2.31 |
| 73 | 1.159 | L-Valine | C_5_H_11_NO_2_ | 117.0795 | -1.41 |
| 74 | 3.695 | Luteone 7-glucoside | C_26_H_28_O_11_ | 516.1654 | -1.69 |
| 75 | 3.386 | Quercetin | C_15_H_10_O_7_ | 302.0439 | -0.72 |
| 76 | 3.038 | Hydrouracil | C_4_H_6_N_2_O_2_ | 128.0591 | -5.01 |
| 77 | 1.157 | Pyridoxal | C_8_H_9_NO_3_ | 167.0590 | -4.49 |
| 78 | 1.930 | 3-Indoleacetic Acid | C_10_H_9_NO_2_ | 175.0641 | -5.27 |
| 79 | 3.487 | Phenylacetic acid | C_8_H_8_O_2_ | 136.0531 | -3.41 |
| 80 | 0.922 | L-Histidine | C_6_H_9_N_3_O_2_ | 155.0701 | -1.93 |
| 81 | 3.576 | Naringenin-7-O-Glucoside | C_21_H_22_O_10_ | 434.1231 | -1.02 |
| 82 | 6.117 | Liquiritigenin | C_15_H_12_O_4_ | 256.0746 | -1.69 |
| 83 | 5.360 | Glabrolide | C_30_H_44_O_4_ | 468.3260 | -0.58 |
| 84 | 3.384 | Genistin | C_21_H_20_O_10_ | 432.1076 | -2.71 |
| 85 | 3.562 | Gentisin | C_14_H_10_O_5_ | 258.0540 | -0.69 |
| 86 | 1.151 | L-Nicotine | C_10_H_14_N_2_ | 162.1164 | -1.85 |
| 87 | 1.164 | Histamine | C_5_H_9_N_3_ | 111.0803 | -2.85 |
| 88 | 1.154 | Niacinamide | C_6_H_6_N_2_O | 122.0487 | -3.94 |
| 89 | 1.155 | 5'-Deoxyadenosine | C_10_H_13_N_5_O_3_ | 251.1033 | -2.72 |
| 90 | 3.311 | 4-Hydroxybenzaldehyde | C_7_H_6_O_2_ | 122.0375 | -3.54 |
| 91 | 1.161 | Benzoic acid | C_7_H_6_O_2_ | 122.0375 | -1.50 |
| 92 | 3.139 | p-Coumaroyl quinic acid | C_16_H_18_O_8_ | 338.1020 | -1.05 |
| 93 | 3.270 | Puerarin | C_21_H_20_O_9_ | 416.1131 | -2.44 |
| 94 | 1.130 | α-ketoisovaleric acid | C_5_H_8_O_3_ | 116.0481 | -2.15 |
| 95 | 1.393 | 2-Furoic acid | C_5_H_4_O_3_ | 112.0168 | -1.82 |
| 96 | 2.907 | Caprolactam | C_6_H_11_NO | 113.0848 | -2.95 |
| 97 | 0.996 | Uracil | C_4_H_4_N_2_O_2_ | 112.0281 | -0.57 |
| 98 | 1.036 | Valeric acid | C_5_H_10_O_2_ | 102.0689 | -2.07 |
