# Supplementary material for: Comparison of Chemical Constituents in Pseudostellariae Radix with Different Dosage Forms Based on HPLC-Q-Exactive Orbitrap/MS Combined with Multivariate Statistical Analysis
Source: Evid Based Complement Alternat Med. 2021 May 8;2021:6644127. doi: 10.1155/2021/6644127 (PMC8128553; doi:10.1155/2021/6644127)
Supplement: Supplementary Materials — Table S1. Components identified with significant differences from Pseudostellariae Radix with different dosage forms in the positive ion mode (p < 0.05). Table S2. Components identified with significant differences from Pseudostellariae Radix with different dosage forms in the negative ion mode (p < 0.05). [file 6644127.f1.zip › 6644127.f1/Table S2.docx]

**Table S2 Components identified with significant differences from Pseudostellariae Radix with different dosage form in negative ion mode（p＜0.05）**

| NO. | *t*_R_/min | Compound | formula | *m/z* | Fold change |
| --- | --- | --- | --- | --- | --- |
| 1 | 9.063 | LPA(0:0/16:0) | C_19_H_39_O_7_P | 410.2418 | 4.49 |
| 2 | 3.017 | Apiin | C_26_H_28_O_14_ | 564.1457 | 1.06 |
| 3 | 0.876 | L-Ascorbic acid | C_6_H_8_O_6_ | 176.0314 | 0.31 |
| 4 | 3.261 | 6''-Malonylapiin | C_29_H_30_O_17_ | 650.1461 | 5.43 |
| 5 | 0.842 | Sucrose | C_12_H_22_O_11_ | 342.1146 | 1.04 |
| 6 | 0.827 | Raffinose | C_18_H_32_O_16_ | 504.1667 | 1.09 |
| 7 | 8.322 | LysoPE(0:0/16:0) | C_21_H_44_NO_7_P | 453.2834 | 4.97 |
| 8 | 4.151 | Hesperetin 7-O-glucuronide | - C_22_H_22_O_12_ | 476.1295 | 2.15 |
| 9 | 5.409 | Isoformononetin | C_16_H_12_O_4_ | 268.0724 | 4.88 |
| 10 | 0.904 | Succinic acid | C_4_H_6_O_4_ | 118.0260 | 0.47 |
| 11 | 0.915 | Thymidine | C_10_H_14_N_2_O_5_ | 242.0890 | 0.73 |
| 12 | 3.652 | Diosmin | C_28_H_32_O_15_ | 608.1705 | 3.17 |
| 13 | 4.435 | Formononetin | C_16_H_12_O_4_ | 268.0721 | 6.62 |
| 14 | 1.158 | Uridine | C_9_H_12_N_2_O_6_ | 244.0681 | 0.52 |
| 15 | 0.833 | L-Glutamate | C_5_H_7_NO_4_ | 147.0523 | 1.35 |
| 16 | 10.105 | Linoleic acid | C_18_H_32_O_2_ | 280.2385 | 3.80 |
| 17 | 0.906 | Citric acid | C_6_H_8_O_7_ | 192.0252 | 3.37 |
| 18 | 0.983 | D-Glucarate | C_6_H_10_O_8_ | 210.0356 | 2.49 |
| 19 | 5.620 | (-)-Jasmonic acid | C_12_H_18_O_3_ | 210.1236 | 1.83 |
| 20 | 5.447 | Auxin a | C_18_H_32_O_5_ | 328.2237 | -1.21 |
| 21 | 3.631 | Tropic acid | C_9_H_10_O_3_ | 166.0624 | -1.94 |
| 22 | 4.306 | Tangeritin | C_20_H_20_O_7_ | 372.1196 | -2.89 |
| 23 | 2.491 | Chlorogenic Acid | C_16_H_18_O_9_ | 354.0934 | -0.83 |
| 24 | 2.863 | p-Coumaroyl quinic acid | C_16_H_18_O_8_ | 338.0988 | -3.19 |
| 25 | 4.094 | Taxifolin | C_15_H_12_O_7_ | 304.0568 | -2.21 |
| 26 | 0.892 | 3'-Sialyllactose | C_23_H_39_NO_19_ | 633.2087 | -1.81 |
| 27 | 0.890 | Glutaric acid | C_5_H_8_O_4_ | 132.0416 | -2.92 |
| 28 | 4.312 | Luteolin | C_15_H_10_O_6_ | 286.0462 | -3.36 |
| 29 | 3.785 | Naringenin-7-O-Glucoside | C_21_H_22_O_10_ | 434.1191 | -2.46 |
| 30 | 3.760 | Sebacic acid | C_10_H_18_O_4_ | 202.1194 | -1.55 |
| 31 | 3.625 | Astragalin | C_21_H_20_O_11_ | 448.0983 | -1.23 |
| 32 | 3.589 | 3'',4''-Diacetylcosmosiin | C_25_H_27_NO_11_ | 516.1237 | -3.37 |
| 33 | 4.979 | Glycitein | C_16_H_12_O_5_ | 284.0668 | -1.05 |
| 34 | 6.114 | Liquiritigenin | C_15_H_12_O_4_ | 256.0721 | -1.46 |
| 35 | 5.997 | Chrysin | C_15_H_10_O_4_ | 254.0564 | -3.77 |
| 36 | 6.934 | Genistein | C_15_H_10_O_5_ | 270.0513 | -2.91 |
| 37 | 4.852 | Malvidin | C_17_H_15_O_7_ | 330.0721 | -4.38 |
| 38 | 4.070 | Daidzein | C_15_H_10_O_4_ | 254.0565 | -3.62 |
| 39 | 5.850 | Hexadecanedioic acid | C_16_H_30_O_4_ | 286.2128 | -1.30 |
| 40 | 3.064 | Gallocatechin | C_15_H_14_O_7_ | 306.0756 | -3.31 |
| 41 | 0.904 | Citramalic acid | C_5_H_8_O_5_ | 148.0362 | -0.44 |
| 42 | 5.287 | Traumatic Acid | C_12_H_20_O_4_ | 228.1346 | -3.00 |
| 43 | 2.037 | Pantothenic Acid | C_9_H_17_NO_5_ | 219.1092 | -1.85 |
| 44 | 0.956 | Quinic acid | C_7_H_12_O_6_ | 192.0622 | -2.72 |
| 45 | 4.541 | Hesperetin | C_16_H_14_O_6_ | 302.0770 | -3.23 |
| 46 | 3.064 | Keioside | C_28_H_32_O_16_ | 624.1652 | -0.62 |
| 47 | 4.461 | Abscisic Acid | C_15_H_20_O_4_ | 264.1345 | -1.52 |
| 48 | 4.494 | Eriodictyol | C_15_H_12_O_6_ | 288.0616 | -2.77 |
| 49 | 1.156 | L-Dopa | C_9_H_11_NO_4_ | 197.0674 | -1.85 |
| 50 | 2.751 | Riboflavin | C_17_H_20_N_4_O_6_ | 376.1351 | -2.27 |
| 51 | 0.912 | α-D-Glucose | C_6_H_12_O_6_ | 180.0616 | -1.13 |
| 52 | 3.965 | Nonanedioic acid | C_9_H_16_O_4_ | 188.1031 | -0.97 |
